# Effect of Obesity on Surgical Outcomes of Lumbar Microdiscectomy: A Retrospective Analysis of 525 Patients

**DOI:** 10.7759/cureus.39719

**Published:** 2023-05-30

**Authors:** Marcus Rommelman, Aleeza Safdar, Rouzbeh Motiei-Langroudi

**Affiliations:** 1 Radiology, Lehigh Valley Hospital, Allenstown, USA; 2 Neurosurgery, University of Kentucky, Lexington, USA

**Keywords:** reherniation, re-operation, lumbar microdiscectomy, surgical outcome, obesity

## Abstract

Introduction: Obesity has been implicated in higher rates of intra-operative complications, as well as increased risk for recurrent herniation and re-operation following lumbar microdiscectomy (LMD). However, the current literature is still controversial about whether obesity adversely affects surgical outcomes, especially a higher re-operation rate. In this study, we have compared surgical outcomes such as recurrence of symptoms, recurrence of disc herniation, and re-operation rates in obese and non-obese patients undergoing one segment LMD.

Methods: A retrospective review was conducted on patients undergoing single-level LMD between 2010-2020 at an academic institution. Exclusion criteria included prior lumbar surgery. Outcomes assessed included the presence of persistent radicular pain, imaging evidence of recurrent herniation, and the need for re-operation due to recurrent herniation.

Results: A total of 525 patients were included in the study. The mean±SD body mass index (BMI) was 31.2±6.6 (range 16.2-70.0). The mean follow-up was 273.8±445.2 days (range 14-2494). Reherniation occurred in 84 patients (16.0%), and 69 (13.1%) underwent re-operation due to persistent recurrent symptoms. Neither reherniation nor re-operation was significantly associated with BMI (p = 0.47 and 0.95, respectively). Probit analysis did not show any significant association between BMI and the need for re-operation following LMD.

Conclusion: Obese and non-obese patients experienced similar surgical outcomes. Our results showed that BMI did not adversely affect reherniation or re-operation rate following LMD. If clinically indicated, LMD can be performed in obese patients with disc herniation without a significantly higher re-operation rate.

## Introduction

Lumbar disc herniation (LDH) is one of the leading causes of low back and radicular leg pain [1]. Patients who are refractory to four to six weeks of medical management (including pain medications, physical therapy, and epidural steroid injections) or those with severe neurologic deficits or cauda equina symptoms are generally considered candidates for surgery [1]. The most common surgical procedures for LDH are lumbar microdiscectomy (LMD) or minimally invasive LMD (MIS-LMD).

The decision to proceed with surgery can be a complex one as there are many other factors that must be taken into consideration. One crucial consideration is the patient’s body habitus. Obesity imposes certain challenges to surgery in general, and spine surgery specifically [2-7]. Obesity has long been recognized as a risk factor for negative outcomes after spine surgery, such as a greater risk of wound infection, more blood loss, longer operation time, and an increased risk of deep venous thrombosis (DVT), recurrence of LDH and re-operation [2-8]. 

The relationship between body habitus and successful surgical management of LDH has been controversial. Data on surgical outcomes of LMD in obese patients are limited with conflicting results. Although recurrence of LDH has been shown to be higher in obese and overweight patients after LMD [8-11], some studies have found that obese individuals experience functional improvement (improvement in back and leg pain) after LMD to the same extent as non-obese patients [12]. Moreover, others have shown that obesity is not a predictor of recurrent intervertebral disc prolapse after LMD [13]. Furthermore, obesity is becoming a universal epidemic, and healthcare professionals are dealing with the issue more than ever. It has been projected that by 2030, nearly one in two adults in the United States (US) will be obese and the prevalence will be higher than 50% in 29 states and not below 35% in any state [14]. As a result, surgeons are facing an increasing number of obese patients requiring surgical interventions, including LMD.

We conducted a retrospective study on the outcomes of single-level LMD for LDH. We specifically compared surgical outcomes including recurrence of symptoms, recurrence of disc herniation, and re-operation rates in obese and non-obese patients undergoing one segment LMD.

## Materials and methods

This is a retrospective study including patients with a diagnosis of LDH undergoing single-level LMD at an academic center, University of Kentucky Albert Chandler Hospital, Lexington, Kentucky, United States, between January 2010 till December 2020. Eligible patients were identified through medical and billing records. University of Kentucky Institutional Review Board approval was obtained for the study (approval number: 62419).

Inclusion and exclusion criteria

Adult patients (18-83 years old) with a diagnosis of lumbar disc herniation (confirmed by lumbar MRI without contrast or a CT myelogram) who had undergone a single-level LMD were included. Patients who had a history of any previous lumbar spine surgery (micro-discectomy, laminectomy, fusion, etc.), any surgery other than single-level LMD, and those who had no follow-up were excluded from the study. Patients with any other procedure performed (bilateral procedure, full laminectomy, MIS discectomy, etc.) were also excluded.

Research variables 

The surgical procedure in all patients included a standard open unilateral laminotomy, medial facetectomy, foraminotomy, and microscopic discectomy. The relevant medical and imaging records were reviewed, and the following variables were recorded: age, sex, body mass index (BMI), indication for surgery, level of LDH, follow up-length, clinical outcome at last follow-up, including recurrence of symptoms (ROS), recurrence of LDH confirmed by imaging (rLDH), and need for re-operation, as well as complications such as post-operative infection and cerebrospinal fluid leak.

Statistical analysis

All data were analyzed with IBM SPSS Statistics for Windows, Version 28.0 (Released 2021; IBM Corp., Armonk, New York, US). Chi-square and independent t-test tests were used to analyze qualitative and quantitative data, respectively. Multivariate regression analysis was used to predict the effect of independent variables on surgical outcomes. P-values < 0.05 were considered statistically significant.

## Results

A total of 525 patients were included in our study after meeting our inclusion criteria. Descriptive characteristics are shown in Table 1.

**Table 1:** Descriptive statistics of study population

Variables	Mean±SD (range)
Age, (year-old)	46.5±14.3 (18-83)
Body mass index (BMI), (kg/m^2^)	31.2±6.6 (16.2-70.0)
Follow-up length, (days)	273.8±445.2 (14-2494)
	Number (%)
Recurrence of radicular symptoms	251 (47.7%)
Recurrence of disc herniation	84 (16%)
Re-operation due to disc herniation	69 (13.1%)

Effect of predictors on surgical outcomes (ROS, rLDH, and re-operation)

The effect of predictors on surgical outcomes (ROS, rLDH, and re-operation) is shown in Table 2 and Table 3. A total of 251 patients (47.7%) experienced ROS. Age and follow-up length significantly predicted ROS (p = 0.01 and <0.01, respectively). Moreover, rLDH, as confirmed by a postoperative MRI or CT myelogram, occurred in 84 patients (16.0%), with the majority occurring at the L5-S1 level (45 patients) or L4-L5 level (33 patients). Follow-up length significantly predicted rLDH (p < 0.001). Sixty-nine patients (13.1%) underwent re-operation due to persistent recurrent symptoms with a confirmed imaging finding. Among these, 29 happened at L4-L5 and 32 at L5-S1 level. Again, follow-up length significantly predicted re-operation (p <0.001). Multivariate regression analysis showed that there was not a significant correlation between any of the independent variables and ROS. Age, gender, BMI, and BMI class did not influence ROS outcome (p = 0.11, 0.51, 0.38, 0.41, respectively). Regarding rLDH, again age, gender, BMI, and BMI class did not influence outcome (p = 0.63, 0.12, 0.52, 0.43, respectively). Lastly, none of the variables influenced re-operation outcome (p = 0.89, 0.09, 0.33, 0.64, respectively).

**Table 2:** Effect of quantitative factors on surgical outcome. ROS: recurrence of symptoms, rLDH: recurrent lumbar disc herniation Each cell represents mean±SD; * denotes statistical significance.

	ROS		p-value	rLDH		p-value	Re-operation		p-value
	No	Yes		No	Yes		No	Yes	
Age (year-old)	48.0±15.4	44.8±12.8	0.01*	46.7±14.5	45.4±13.0	0.43	46.5±14.4	46.4±13.9	0.99
BMI (kg/m^2^)	31.1±6.4	31.2±6.9	0.84	31.3±6.8	30.7±5.8	0.47	31.2±6.5	31.1±7.5	0.95
Follow-up length (days)	66.6±183.6	500.0±529.2	<0.001*	210.8±391.4	604.3±554.5	<0.001*	234.3±424.7	534.5±490.6	<0.001*

**Table 3:** Effect of qualitative factors on surgical outcome. ROS: recurrence of symptoms, rLDH: recurrent lumbar disc herniation. Each cell represents percentage of people experiencing the adverse outcome (ROS, rLDH, Re-operation) within each group; * denotes statistical significance.

	ROS	p-value	rLDH	p-value	Re-operation	p-value
Gender		0.30		0.14		0.10
Male	46.3%		14.1%		11.3%	
Female	51.0%		19.0%		16.2%	
Level		0.04*		0.72		0.90
L1-L2	28.6%		14.3%		14.3%	
L2-L3	85.7%		0.0%		0.0%	
L3-L4	33.3%		11.1%		15.6%	
L4-L5	45.7%		15.7%		13.8%	
L5-S1	51.8%		17.6%		12.5%	
Surgical site complication		0.34		0.25		0.43
No	47.5%		15.7%		12.9%	
Yes	60%		26.7%		20%	

Effect of BMI on surgical outcomes (ROS, rLDH, and re-operation)

BMI was not different in those experiencing rLDH compared to those who did not (30.7±5.8 vs. 31.3±6.8, respectively; p = 0.47). Furthermore, BMI was not different in those undergoing re-operation compared to those who did not (31.2±6.5 vs. 31.1±7.5, respectively; p = 0.95). Probit analysis did not show any significant association between BMI and the need for re-operation (Probit model: re-operation probability = -1.098 + BMI x -0.001; p = 0.95). We further classified BMI into the seven standard categories (underweight: BMI < 20.0; normal weight: BMI between 20.0-24.9; overweight: 25.0-29.9; obese grade 1: 30.0-34.9; obese grade 2: 35.0-39.9; obese grade 3: 40.0-49.9; obese grade 4: BMI equal or more than 50.0). Analysis showed that BMI class did not influence ROS, rLDH, or re-operation (p = 0.64, 0.49, 0.66, respectively). Results are shown in Table 4. 

**Table 4:** Effect of BMI class on surgical outcome. ROS: recurrence of symptoms, rLDH: recurrent lumbar disc herniation. Each cell represents percentage of people experiencing the adverse outcome (ROS, rLDH, Re-operation) within each group

BMI class	ROS	rLDH	Re-operation
<20.0	83.3%	33.3%	33.3%
20.0-24.9	44.2%	11.7%	10.4%
25.0-29.9	46.4%	18.5%	14.3%
30.0-34.9	49.6%	17.0%	13.5%
35.0-39.9	45.5%	13.0%	13.0%
40.0-49.9	50.0%	10.9%	8.7%
>50.0	33.3%	33.3%	0.0%

## Discussion

Obesity has nearly tripled worldwide during the past five decades and it has been projected that by 2030 nearly 50% of the US population will be obese [14]. As a result, surgeons in all specialties and subspecialties are facing an increasing number of obese patients requiring surgical interventions. Obesity has been reported as a predictor for negative outcomes, such as wound infection, more blood loss, longer operation time, and increased risk of deep vein thrombosis (DVT), after spine surgery but clinical outcomes have been reported to be similar between obese and non-obese patients undergoing spine surgery [2, 4]. In one study it has been reported that obese patients were 12 times more likely to have rLDH and 30 times more likely to require re-operation compared to non-obese patients [8]. However, this study had a small sample size of 75 patients only [8]. In our study, we did not find any significant association between BMI and the main surgical outcome measures (ROS, rLDH, or re-operation). We implemented different statistical methods (comparing the mean BMI in those who had an adverse outcome vs. those who didn’t, classifying the BMI into levels, and probability analysis), all confirming the same results. There was a significant association between our outcome measures (ROS, rLDH, and re-operation) and length of follow-up. Patients who were followed for a longer period were more likely to manifest adverse outcomes, either because patients with recurrent symptoms/herniation were more prone to seek medical care, or adverse outcomes (ROS or rLDH) were more likely to be diagnosed when patients were followed up for longer. It can be suggested that despite all the known technical challenges and increased risk of medical complications of performing surgeries in obese patients, LMD can still be performed in this group without significantly higher risk for surgical complications such as ROS, rLDH, and re-operation. This is consistent with the results of Rihn et al., who have reported that even though obese patients had a significantly greater operative time, intra-operative blood loss, and length of hospital stay and less improvement in different patient-reported outcome measures (PROM) compared to non-obese patients, outcomes such as rLDH and need for re-operation did not differ significantly between obese and non-obese patients, in this trial, which is consistent with our results [4]. Another study has reported that obese individuals experienced functional improvement after LMD to the same extent as non-obese patients based on improvements in Oswestry Disability Index (ODI), Euro quality of life 5 dimension (EQ-5D), and back pain numeric rating, and leg pain numeric rating, which were comparable between the two groups. However, rLDH and re-operation rates were not reported in this study [12].

Our study has a few limitations. Firstly, we were looking at the data retrospectively, and the possibility of selection bias cannot be completely ruled out. More important, there was a large variability in follow-up length in our patient population ranging from a minimum follow-up of two weeks to up to seven years. It is hard to compare the more critical recurrent rates of surgery of patients with two-week and seven-year follow-up. Lastly, we focused primarily on surgical outcomes including ROS, rLDH, and re-operation, but functional patient-reported outcomes were not reported in our study. In contrast, our study had a large sample size of 525 patients, which is one of the largest reported to date for surgical outcomes of LMD in obese patients. We suggest that prospective studies with longer and pre-set follow-ups should be conducted to provide a deeper understanding of outcomes after LMD in obese vs. non-obese patients.

## Conclusions

Our study showed that BMI did not negatively predict rLDH or re-operation rate following single-level LMD. The results suggest that if clinically indicated, LMD can be performed in obese patients with disc herniation without significantly higher risk for rLDH or re-operation. We suggest that prospective studies with longer and pre-set follow-ups should be conducted to provide a deeper understanding of outcomes after LMD in obese vs. non-obese patients.
